# Antibody to Heat Shock Protein 70 (HSP70) Inhibits Human T-Cell Lymphoptropic Virus Type I (HTLV-I) Production by Transformed Rabbit T-Cell Lines

**DOI:** 10.3390/toxins4100768

**Published:** 2012-09-26

**Authors:** Hanan Fallouh, Wahib Mahana

**Affiliations:** 1 Faculty of Science, University of Damascus, Damascus, Syria; Email: hafa2@live.fr; 2 Université de Bretagne Occidentale & Université Paris Sud, Endotoxines, Bat: 409, IGM, UMR 86216, 91405 Orsay cedex, France

**Keywords:** HTLV-I, leukemogenesis, heat shock proteins, antibodies, animal model

## Abstract

Adult T cell leukemia is a fatal malignant transformation caused by the human T-cell lymphoptropic virus type I (HTLV-I). HTLV-I is only associated with the development of this disease in a small percentage of infected individuals. Using two rabbit transformed T-cell lines; RH/K30 (asymptomatic) and RH/K34 (leukemogenic), we have investigated the expression of heat shock proteins (HSP) 90 and 70 and the role of anti-HSPs antibodies on virus production. HSPs surface expression was higher on RH/K34 than RH/K30 cells. Heat treatment of cells increased the expression of HSPs proteins and virus production; HSPs augmentation was stabilized after 12 h and virus production reached a maximum between 8 h–12 h then returned to normal level after 24 h of culture. Incubation of cells only with rabbit anti-HSP 70 antibodies prevented virus production specifically in the leukemogenic cell line. The results indicate a relationship between HSP 70 and virus production.

## 1. Introduction

Heat shock proteins (HSPs) are a conserved family of proteins that are constitutively expressed in virtually all nucleated cells. Their synthesis is enhanced in response to environmental stress and may have important physiological or pathological implications [1] HSPs are considered as a cancer associated antigen [2]. They are overexpressed in a wide range of cancers and are implicated in tumor cell proliferation, differentiation, invasion, metastasis, death, and recognition by the immune system [3]. Moreover, aberrant extracellular and dendritic cell surface expression of HSP 70 was observed in tissues from specimen with autoimmune diseases [4].

Evidence that HSP 70 may be involved in the assembly of virions has been provided by Macejak and Luftig [5], who reported that HSP 70 is associated with the fiber protein of adenovirus and by Macejak and Sarnow [6] who reported its association with the capsid precursor P1 of both poliovirus type 1 and coxsackievirus B1 in infected human cells.

Human T-lymphotropic virus type I (HTLV-I) is the etiological agent of adult T-cell leukemia/lymphoma (ATLL) and HTLV-I associated myelopathy/tropical spastic paraparesis (HAM/TSP). HTLV-I is only associated with the development of one of the several chronic diseases cited above in a small percentage of infected individuals while the majority remains asymptomatic [7]. Mechanisms by which infection with this retrovirus may result in such diverse outcomes are not well understood. 

HTLV-I readily infects rabbits and, in some specific instances, infection leads to disease in experimental animals [8,9,10]. Although inoculation with the majority of HTLV-I transformed rabbit cell lines gives rise to chronic asymptomatic infection, administration of certain HTLV-I infected T cell lines leads to acutely fatal disease that mimics human ATLL [11,12,13].

RH/K30 and RH/K34 are two HTLV-I transformed T cell lines; RH/K30 leads to asymptomatic infection while RH/K34 causes a fatal leukemia-like disease accompanied by thymic depletion via apoptosis [11]. Despite the overwhelming differences in their *in vivo* behavior, these two T cell receptor γδ T cell lines have only minor differences in expression of surface markers. The integrated proviruses differ by only 18 nucleotides of their 9 kb sequence with identical tax and envelope proteins [9,13]. It has been reported that thermal stress responses enhance HTLV-I genes and proteins expression [14,15,16]. In the rabbit model, anti-HSP auto-antibodies were reported in the sera of HTLV-I infected rabbit and rabbit having high titer of anti-HSP antibodies can overcome challenge with the leukemogenic cell line RH/K34 [17]. To further understand the relation between stress proteins and HTLV-I infection in the rabbit model, the expression of HSP on the surface of HTLV-I transformed cell line RH/K30 and RH/K34 were tested, and then cells were incubated at 42 °C for different times with or without antibodies to HSPs (70 and 90). Augmentations of the expression of HSP as well as virus production were observed during heat treatment. And antibody to HSP 70 prevents virus production. Our results indicate that HSP 70 may play a modulating role on virus production during stress conditions. 

## 2. Results and Discussion

### 2.1. Expression of HSP on Cell Surface and Response to Heat Shock Treatment

**Figure 1:**
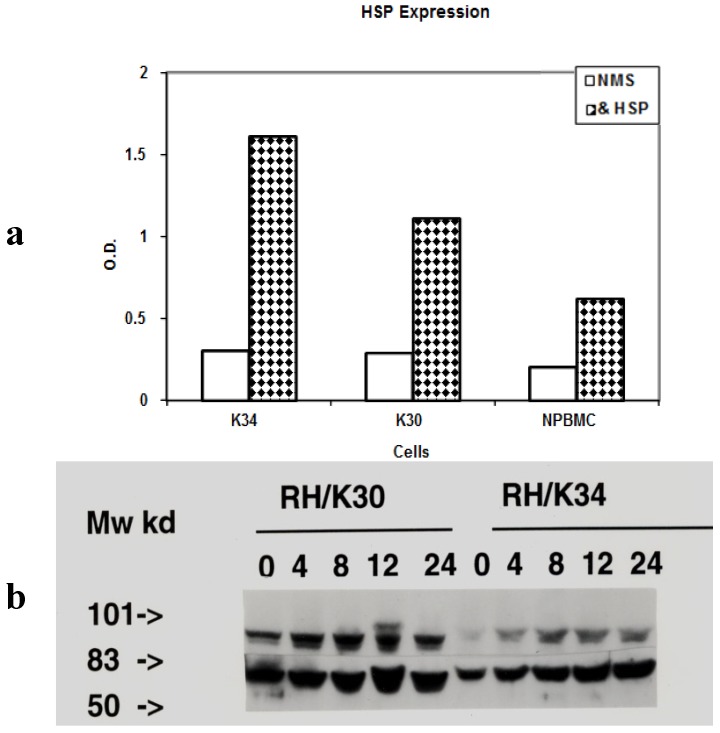
Expression of HSP on HTLV-I transformed rabbit cells. (**a**) Expression of HSP at the surface of rabbit cells measured by ELISA indirect test. HTLV-I transformed rabbit cell lines; leukemogenic RH/K34 (K34), asymptomatic cell line RH/K30 (K30) and normal rabbit peripheral blood mononuclear cell (NPBMC) were incubated in V bottom plate either with mouse anti-HSP antibodies (& HSP) or normal mice sera (NMS) then reveled by peroxidase labeled goat anti-mouse Ig; (**b**) Immunoblot analysis with rabbit anti-HSP 70 and anti-HSP 90 antibodies of whole cell lysates from RH/K30 and RH/K34 cell line samples harvested at different times (0 h–24 h) after exposure to heat treatment at 42 °C. Cell lysates were separated by SDS/PAGE on a 10% gel and transferred onto Immobilon P membrane. Blots were developed either with rabbit anti-HSP 90 antibodies (Upper line), or with rabbit anti-HSP 70 antibodies (Lower line) and peroxidase labelled goat anti-rabbit Ig. Experiment was repeated three times and the figure represents results of one representative test.

The presence of HSP on the surface of the two HTLV-I rabbit cell lines RH/K30, RH/K34 and the rabbit normal peripheral blood mononuclear cells (PBMC) was detected using mouse anti-HSP antibodies. The result, presented in Figure 1a, showed that the HTLV-I transformed cells expressed more HSPs at their surface than normal cells. RH/K30 and RH/K34 expressed respectively about two and three times more HSPs that normal PBMC.

**Figure 2:**
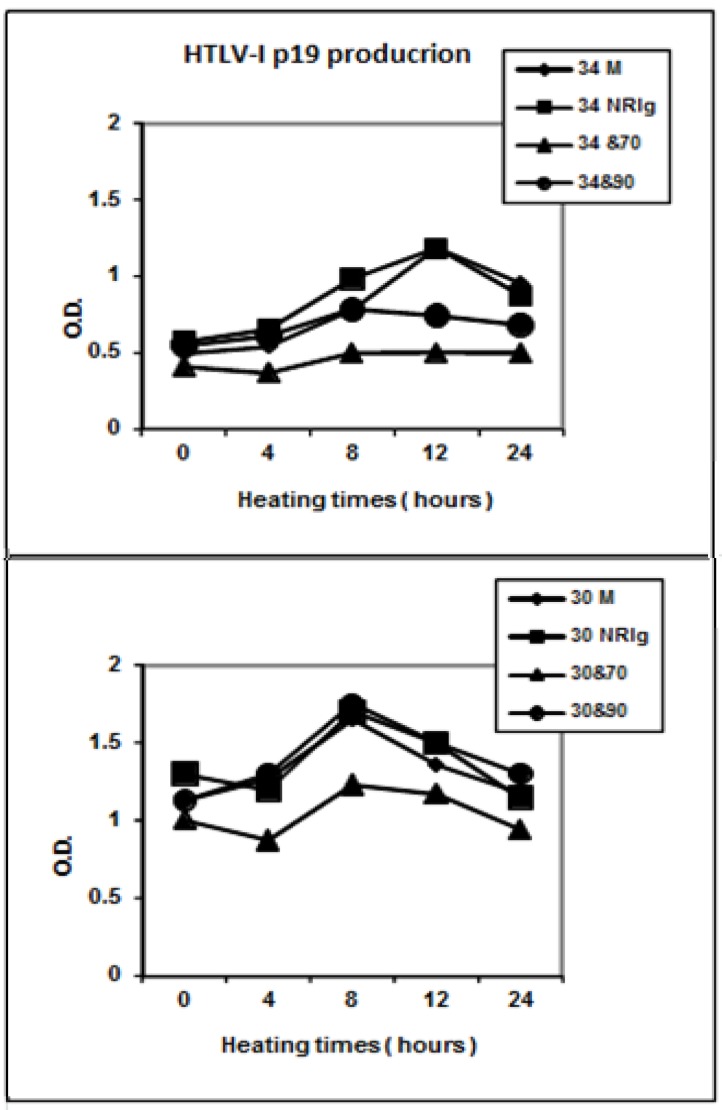
Modulation of HTLV-I production by anti-HSP 70 antibodies. HTLV-I rabbit transformed cell lines RH/K34 (34) and RH/K30 (30) were incubated at 42 °C during indicated times either with medium alone (M), normal rabbit Ig (NRIg), rabbit anti-HSP 70 antibodies (&70) or with rabbit anti-HSP 90 antibodies (&90). Then the supernatant were tested for the presence of p19 antigen by ELISA test according to the manufactory indication.Experiment was repeated three times and the figure represents results of one representative test.

HTLV-I transformed cells RH/K30 and RH/K34 were first placed at 42 °C in presence of 5% CO_2_ for different times. The viability of cells was monitored by the incorporation of trypan blue and no significant difference was observed between heated and non heated cells. Washed cells were treated with lysis buffer, fractionated on SDS gel, transferred to PVDF membrane and blotted either with anti-HSP 70 or anti-HSP 90 antibodies. Results presented in Figure 1b indicated that the level of both HSPs increased during the first 12 h of treatment, and then stabilized at high level until 24 h. However, in RH/K34 cell line a difference could be noted between the strong expression of HSP 70 as opposed to the weaker expression of HSP 90. When the level of virus p19 antigen was monitored in the cell supernatants, we found that the amount of p19 was increased during heat treatment, reaching a maximum at 8 h for the asymptomatic cell line RH/K30 and 12 h for the leukemogenic one RH/K34, before a return to normal basic levels after 24 h of treatment, see Figure 2. The difference concerning the time effect on the cell response to heat treatment may be due to the amount of the virus produced by the cells. In fact RH/K30 produces more virus than RH/K34 (data not shown).

### 2.2. Role of Anti-HSP 70 Antibodies on Virus Production

In a further set of experiments, cells were incubated with the medium alone or in the presence of either normal rabbit IgG, rabbit anti-HSP 90 IgG, or rabbit anti-HSP 70 IgG. No difference was observed for the levels of HSPs expression (data not shown). Inversely, when we tested the amount of viral p19 antigen produced by the cells after the different treatments, we found that the p19 level in the supernatant of leukemogenic cell line RH/K34 was significantly reduced but only in presence of rabbit anti-HSP 70 antibodies (Figure 2).

Immunoprecipitation of HSP 70 from the RH/K34 cell line using the anti-HSP 70 antibodies led in few but not all tests, to co-precipitate a molecule of 20 kDa, reacting with antibody to the HTLV-I glycoprotein gp46 (data not shown). The identity of this molecule remains undetermined. It could be a part of gp46 because it has been reported that a peptide from gp46 interacts specifically with a member heat shock protein family 70 [18]. 

The results presented here show that HSPs are strongly expressed on the surface of HTLV-I transformed cells and the leukemogenic cell line RH/K34 expressed them more than the non-leukemogenic cell line RH/K30. These results are in agreement with those showing that in different cancers and in autoimmune diseases cells can express a high level of HSPs on their surface [3,4,19].

The expression of HSPs was increased after heat treatment in both cell lines over the 24 h of treatment. Expression was progressively increased until 12 h, and then seems to be stabilized at high levels to confer protection to the cells. In fact, a non-significant difference was found between the number of dead cells before and after the heat treatment which could be related to the effect of HSP. 

In parallel to the augmentation of HSPs expression, the virus production by the cells monitored by the expression of p19 protein was also increased during the first 8–12 h of culture, after this time and inversely to the HSP expression, it returned to the basic level after 24 h. A comparable result for the production of the HTLV-I p24 protein was reported by Andrews *et al.* [14] with a little difference concerning the kinetic of the augmentation of p24 which may be due to difference in the origins of the cells and the tested protein. Moreover, addition of specific rabbit anti-HSP 70 antibodies to the cell culture can prevent virus production. Inhibition of virus production was more pronounced and significant in the leukemogenic RH/K34 cell line than in the asymptomatic RH/K30 cell line. This may have resulted first from the high expression of HSP 70 in RH/K34 comparing to that in HSP 90 and second by the high virus production by RH/K30 cells comparing to that by RH/K34. The mechanisms by which anti-HSP 70 antibodies exert such inhibitory effect remain unknown. HSP 70 could interact with viral elements involved in virus production and its blockage by specific antibodies could interfere with virus production. In fact, it has been reported in the canine distemper virus model that antibody to HSP 72 suppressed both basal and stress-enhanced polymerase activity leading to the suppression of cytopathic effect linked to the stress [20]. In another study on influenza virus, it was shown that HSP 70 interacts with the ribonucleoprotein complex and inhibits its nuclear export leading to the inhibition of virus production [21]. We and other reported the presence of natural auto anti-HSP antibodies in the sera of normal and retrovirus infected human and animals [22,23,24,25] but little is known concerning their role. 

It has been hypothesized that there are relationships between the expression of HSP on tumor cells, the immune response and the prognosis of tumors. Cells expressing high level of HSP activate immune response that leads to their elimination and only the tumor clones with low HSP expression are able to evade an immune response and invade other tissues [26]. In agreement with this, the asymptomatic cell line RH/K30, which expresses less HSP than the leukemogenic cell line RH/K34, can easily infect rabbit but without any pathogenic effect. The viral molecular clone corresponding to the virus from this line was more infective than the molecular clone from RH/K34 [13]. On the other hand, the rabbit reaction to the infection by the leukemogenic cell line RH/K34 that has higher surface HSP expression suggests a more ambiguous relationship between the expression of HSP and cancer cells invasion. Instead of having a strong and protective immune response induced by the high expression of HSP on the leukemogenic cell, this cell kills the rabbit. This result may suggest that the rabbit death could probably be due to a strong auto-immune response induced by this cell line. In agreement with this, we have observed that RH/K34 cell line increases multiplication in presence of cyclosporin A (data not shown) which is involved in some cases in the induction of autoimmunity [27]. Furthermore, a hyper reactivity of immune system that can induce autoimmunity has been reported in HTLV-I transgenic mice [28]. The time of the rabbit reaction after injection by RHK/34 cell line is too short (one to two weeks) to allow detailed investigation of the immune response, however, studies on dead rabbits showed the presence of apoptotic cells in their thymus [11].

Augmentation of virus production by heat treatment could play a role in the interruption of latency during HTLV-I infection. However, this modulation is quick and less durable comparing to HSP increase which may also activate the immune system. In agreement with our data a correlation between the induction of HSP 70 and enhanced viral reactivation was reported in mammalian cells [29].

The study provides further insights on the role of HSPs in HTLV-I infection, latency and immune response. Interactions virus/HSPs may depend on the kind of infection (leukemogenic versus asymptomatic), the level of HSP expression and the stress reaction which may be caused by different factors. The results indicate a complicate relation between HSP expression, HTLV-I infection and the role of anti-HSP 70 antibodies on virus production.

## 3. Experimental Section

### 3.1. Cell Lines and Treatment

The rabbit γδ T cell lines, RH/K30 and RH/K34, were obtained by transformation *in vitro* by HTLV-I from irradiated human MT-2 T cell line [9]. Rabbit normal PBMC were purified by classical method on Ficoll gradient. Cells were maintained in culture in RPMI complete medium containing 10% fetal calf sera (PAA Laboratories, Les Mureaux, France), 2 mM L-glutamine, 100 U/mL penicillin and 100 µg/mL streptomycin.

For stress treatment, cells were cultured in same medium with or without anti-HSP antibodies at 42 °C for different times. At indicated times; cells were harvested by centrifugation, the supernatant were kept frozen for following tests and the cells were washed with phosphate-buffered saline (PBS) and frozen until use. Anti-HSP antibodies were diluted in medium and used at indicated concentrations. Each test was repeated at least three times and results from typical test were presented.

### 3.2. Antibodies to Heat Shock Proteins

Bovine heat shock protein 70 and 90 were purchased from Stressgen Biotechnologies (Victoria, BC, Canada). Mice were immunized with 25 µg of a mixture HSP 70 and 90 incorporated in complete Freund’s adjuvant for the first time and thirty days later with the same amount of HSP incorporated in incomplete Freund’s adjuvant. Immunization was repeated three times. Blood samples were collected one week after the second and further immunizations from the retro‑orbital plexus and sera were stored at −20 °C until use. 

For rabbit anti-HSPs antibodies, New Zealand White rabbits were immunized with 50 µg of either HSP 70 or HSP 90 incorporated in complete Freund’s, and incomplete Freund’s adjuvant for following Immunization. 

Polyclonal antibodies were tested on the HSP 70 and HSP 90, and the good serums were pooled and kept to use in further experiments. All animal experiments have been done in accordance with institutional ethic guidelines.

Rabbit antibodies were purified on protein G column (Pierce, Thermo Scientific, Brebières, France) as described by the manufactories, filtered on 0.22 µm membrane filter, frozen and stored until use in cell culture experiment.

### 3.3. Enzyme-Linked Immunosorbent Assay (ELISA)

Three ELISA tests were used in the work. The first was to detect HSP at the cell surface; V bottom plates were saturated by overnight incubation with PBS containing 10% calf fetal serum and 0.2% of sodium azide. After washing with PBS, 10^5^ cells were distributed in each well and incubated for two hours with 200 µL of the same PBS. After centrifugation for 10 min at 1500 rpm, the plate was quickly and gently turned up side down to remove the supernatant then incubated with 100 µL/well of mouse anti-HSP antibodies for one hour at room temperature. After three washes, with 200 µL of PBS, plates were incubated with peroxidase labeled goat anti-mouse Ig for one hour at room temperature. After washing, the 2,2'-azino-bis[3-ethylbenzothiazoline-6-sulfonic acid] (ABTS) substrate was added and incubated for 30 min with the cells. Then the supernatant was removed to flat bottom plate and the optical density was measured at 405 nm. 

Second was to test the anti-HSP antibodies activity in mice and rabbits sera. Flat bottom plates coated overnight at 4 °C with 1 µg/mL of HSP proteins in 100 µL of carbonate-bicarbonate buffer (0.1 M, pH 9.6) were washed with phosphate-buffered saline-Tween 20 (PBS-T) (0.05%; *v*/*v*) and blocked for one hour at 37 °C with PBS-T containing 0.5% gelatin (PBS-T-G). Then diluted sera in the same buffer were added. After one hour of incubation at 37 °C and four washes, corresponding peroxidase labeled goat anti-Ig antibodies were added and the plates were incubated for another hour at 37 °C. After four washes, ABTS substrate was added and then plate were read at 405 nm with a Titertek Multiscan instrument (Skatron, Oslo, Norway). 

Third was to quantify the amount of HTLV-I p19 antigen in the supernatant of transformed cells. An ELISA kit was purchased from ZeptoMetrix Corporation, (Buffalo, NY, USA) and used as described by the manufactories. All of ELISA tests were repeated at least three times and results from a typical test were presented.

### 3.4. Western Blotting

PBS washed cells were treated with lysis buffer (10 mM Tris-HCL PH 7.5, 150 mM NaCl, 1% Triton-X 100, 0.02% NaN_3_, 1 mM PMSF, 10 mM Nα-Tosyl-Lys Chloromethyl Ketone (TLCK), 5 mM iodoacetamide, 2 mM aprotinin and 2 mM leupeptin) for one hour at 4 °C then centrifuged for 15 min at 3000 rpm to discard non soluble fraction. Soluble cell lysates were fractionated on SDS gel, transferred to PVDF membrane and blots were developed either with anti-HSP 70 or anti- HSP 90 antibodies then with peroxidase labeled goat anti-Ig rabbit antibodies, using the enhanced chemiluminescence substrate (ECL) Amersham, (Piscataway, NJ, USA). 

### 3.5. Statistical Analysis

Student’s *t*-test was used to compare the values of obtained results.

## 4. Conclusions

We can conclude from our data that heat treatment of HTLV-I infected cell modulates HSP 70 and 90 expressions and virus production. HSPs expression remained strong during the 24 h of treatment, inversely, virus production increased during the first 12 h of treatment then attempted to return to a normal level. Addition of anti-HSP 70 antibodies prevents virus production. HSP 70 may interact with HTLV-I through a mechanism involved in the virus production. The result of this interaction is more evident in the leukemogenic infected cell than in the asymptomatic infected cell.
